# Do neural correlates of face expertise vary with task demands? Event-related potential correlates of own- and other-race face inversion

**DOI:** 10.3389/fnhum.2013.00898

**Published:** 2013-12-24

**Authors:** Holger Wiese

**Affiliations:** DFG Research Unit Person Perception, Institute of Psychology, Friedrich Schiller University of JenaJena, Germany

**Keywords:** faces, event-related potentials, N170, own-race bias, inversion

## Abstract

We are typically more accurate at remembering own- than other-race faces. This “own-race bias” has been suggested to result from enhanced expertise with and more efficient perceptual processing of own-race than other-race faces. In line with this idea, the N170, an event-related potential correlate of face perception, has been repeatedly found to be larger for other-race faces. Other studies, however, found no difference in N170 amplitude for faces from diverse ethnic groups. The present study tested whether these seemingly incongruent findings can be explained by varying task demands. European participants were presented with upright and inverted European and Asian faces (as well as European and Asian houses), and asked to either indicate the ethnicity or the orientation of the stimuli. Larger N170s for other-race faces were observed in the ethnicity but not in the orientation task, suggesting that the necessity to process facial category information is a minimum prerequisite for the occurrence of the effect. In addition, N170 inversion effects, with larger amplitudes for inverted relative to upright stimuli, were more pronounced for own- relative to other-race faces in both tasks. Overall, the present findings suggest that the occurrence of ethnicity effects in N170 for upright faces depends on the amount of facial detail required for the task at hand. At the same time, the larger inversion effects for own- than other-race faces occur independent of task and may reflect the fine-tuning of perceptual processing to faces of maximum expertise.

## Introduction

Humans can typically recognize an immense number of previously seen faces and are therefore often considered to be experts in face recognition. Importantly, however, such expertise considerably varies depending on an individual's experience with a specific category of faces. The maybe best known example for this claim is the so-called own-race^1^ bias (Malpass and Kravitz, 1969; Meissner and Brigham, 2001), i.e., the finding that participants are typically more accurate at recognizing faces from their own compared to another ethnic group. This phenomenon has been explained by the substantially larger experience that most people have with faces from their own relative to other ethnic groups, resulting in a fine-tuning of face perception mechanisms (Rossion and Michel, 2011). For instance, it has been found that so-called holistic face processing, i.e., the merging of facial features into a single Gestalt-like representation, but also the processing of the features themselves, is more efficient for own- relative to other-race faces (Tanaka et al., 2004; Rhodes et al., 2006; Hayward et al., 2008).

Neural correlates of face perception have been extensively studied using event-related potentials (ERPs). Most researchers agree that the first component with a high degree of selectively for faces is the N170 (Bentin et al., 1996; Rossion and Jacques, 2008), a negative component peaking at approximately 170 ms at occipito-temporal scalp sites. The N170 is larger for faces than for most objects (e.g., Rossion et al., 2000; Itier and Taylor, 2004), and it has been suggested to reflect the structural encoding (Eimer, 2011) or detection of a face-like pattern (Schweinberger and Burton, 2003; Amihai et al., 2011).

Following-up on the suggestion that the own-race bias results from differences in perceptual face processing, a number of studies examined whether the ethnicity of a face affects the amplitude of the N170. At first sight, the results of these studies are rather discrepant, with roughly half of them reporting no significant difference in N170 for own- vs. other-race faces (James et al., 2001; Caldara et al., 2003, 2004; Wiese et al., 2009; Vizioli et al., 2010a,b; Herzmann et al., 2011; Ofan et al., 2011, 2013; Chen et al., 2013), and the other half finding larger amplitudes for other- relative to own-race faces (Herrmann et al., 2007; Gajewski et al., 2008; Stahl et al., 2008, 2010; Walker et al., 2008; He et al., 2009; Balas and Nelson, 2010; Brebner et al., 2011; Caharel et al., 2011; Wiese, 2012; Montalan et al., 2013; Wiese et al., 2013). Importantly, it has been suggested that varying task demands may contribute to these discrepant findings (e.g., Ito and Bartholow, 2009; Caharel et al., 2011). A larger N170 for other-race faces might occur when the identity of the face is relevant for the task, whereas N170 might be similar for own- and other-race faces when such detailed facial information is not task-relevant (as suggested by Ito and Bartholow, 2009).

Broadly in line with this idea, the publications which I am aware of to date can be roughly assigned to one of three categories (see Table 1): (i) studies with tasks that are based on *superficial information* in which specific facial detail was not directly task-relevant (e.g., orientation tasks, detection of target objects etc.), (ii) studies in which *categorical information* was task-relevant (e.g., race categorization, gender categorization etc.), and (iii) studies in which the encoding of *identity information* of individual faces was necessary to perform the task (e.g., recognition memory, identity-repetition tasks etc.)^2^. As can be seen in Table 1, when studies are split into these categories the results are relatively consistent, with few exceptions from a clear-cut overall pattern: Whereas seven out of nine studies examining identity information reported an N170 ethnicity effect, with larger amplitudes for other- relative to own-race faces, only two out of ten studies using a task based on superficial information reported such an effect. When the task was based on categorical information, four out of six studies observed larger N170 amplitudes for other-race faces, with the remaining two either showing no effect or larger amplitudes for own-race faces. A crosstab χ^2^ test (with studies classified by task category as dependent variables and coding a larger N170 for other-race faces as 1 and all other results as 0) resulted in a significant effect (χ^2^ = 7.016, *p* = 0.030), suggesting a relevant influence of task demands on the occurrence of the N170 ethnicity effect. As is also evident from Table 1, other factors that might potentially contribute to the presence vs. absence of an N170 ethnicity effect (e.g., the use of color vs. grayscale images, or the specific other-race tested) do not offer a similarly straightforward explanation.

**Table 1:** **Previous studies on N170 ethnicity effects, sorted by task categories**.

**Study**	**Participants' ethnic group**	**Other-race faces**	**Images**	**Task**	**N170 Amplitude**	
					**Upright faces**	**Inversion effect**
**SUPERFICIAL TASKS; CATEGORY OR IDENTITY INFORMATION NOT TASK-RELEVANT**						
Caldara et al., 2003	Caucasian	Asian	Grayscale	“Count butterfly stimuli”	Own = other	Not tested
Gajewski et al., 2008	Exp. 1: Caucasian	Asian, African	Grayscale	“Detect door stimuli”	Own < other	No basic effect
	Exp. 2: Asian	Caucasian, African	Grayscale	“Detect door stimuli”	Own = other	No basic effect
Wiese et al., 2009	Caucasian	Asian	Grayscale	“Stimulus upright/inverted?”	Own = other	Own = other
Vizioli et al., 2010a	Asian/Cauc.	African, Asian/Cauc.	Grayscale	“Detect red/green faces”	Own = other	Own > other
Vizioli et al., 2010b	Asian/Cauc.	Asian/Cauc.	Grayscale	“Press if stimulus is inverted”	Own = other	Not tested
Balas and Nelson, 2010	Caucasian	African-American	Color	“Stimulus upright/inverted?”	Own < other	No basic effect
Ofan et al., 2011	82% Caucasian, 18% Asian	African-American	Two-tone	“Target word pleasant/unpleasant?”	Own = other	Not tested
Ofan et al., 2013	Caucasian	African-American	Two-tone	“Target word pleasant/unpleasant?”	Own = other	Not tested
Senholzi and Ito, 2013	Caucasian	African-American	Color	“Detect butterfly stimuli”	Own = other	Not tested
Chen et al., 2013	Asian	Caucasian	Grayscale	“Count flowers”	Own = other	Own = other
**FACE CATEGORY TASK-RELEVANT**						
Caldara et al., 2004	Caucasian	Asian	Grayscale	“Face Asian or European?”	Own = other	Not tested
He et al., 2009	Caucasian	Asian, African-American	Color	“Face female or male?”	Own < other	Not tested
Brebner et al., 2011	Caucasian	African-American	Color	“Face older or younger than 21?”	Own < other	Not tested
Caharel et al., 2011	Caucasian	Asian, African	Color	“Face own- or other-race?”	Own < other	Own > other
Montalan et al., 2013	Caucasian	African	Grayscale	“Face African or Caucasian?”	Own < other	Own > other
Senholzi and Ito, 2013	Caucasian	African-American	Color	“Race same as in last image?”	Own > other	Not tested
**FACIAL IDENTITY TASK-RELEVANT**						
James et al., 2001	Caucasian	Asian	Grayscale	“Face learned or new?”	Own = other	Not differentially tested
Herrmann et al., 2007	Caucasian	Asian	?	“Face same as in last image?”	Own < other	Not tested
Stahl et al., 2008	Caucasian	Asian	Grayscale	“Face learned or new?”	Own < other	Not tested
Walker et al., 2008	Caucasian	African	Grayscale	“Face same as in last image?”	Own < other	Not differentially tested
Stahl et al., 2010	Caucasian	Asian	Grayscale	“Face learned or new?”	Own < other	Not tested
Herzmann et al., 2011	Asian/Cauc.	Asian/Cauc.	Grayscale	“Face learned or new?”	Own = other	Not tested
Wiese, 2012	Caucasian	Asian	Grayscale	“Face learned or new?”	own < Other	Not tested
Senholzi and Ito, 2013	Caucasian	African-American	Color	“Face same as in last image?”	Own < other	Not tested
Wiese et al., 2013	Asian/Cauc.	Asian/Cauc.	Grayscale	“Face learned or new?”	Own < other	Not tested

In a recent study, Senholzi and Ito (2013) directly tested the influence of task on the N170 ethnicity effect. In line with the above-described pattern, they found that ethnicity effects were absent in a butterfly detection task, whereas N170 was larger for other-race faces in an identity task. In a categorization task, however, larger amplitudes for own-race faces were observed. In sum, the systematic review of the literature provided here points to an important contribution of task on the presence vs. absence of the N170 ethnicity effect, but differences between studies may have also resulted from additional factors varying between participant groups (such as varying long-term expertise with other-race people in different countries, see Rossion and Michel, 2011). Moreover, in the study by Senholzi and Ito (2013) task was manipulated as a between-subjects factor, which introduced the possibility that group differences other than task that were not controlled in this study (such as differences in quality or quantity of contact to other-race people, or the distribution of participant gender in the three tasks), might have affected the results.

Furthermore, a number of the above-cited studies examined the so-called face inversion effect (FIE) for own- and other-race faces. It is well established that the picture-plane rotation of a face by 180° substantially impairs its recognition, and this effect is disproportionally stronger for faces relative to other objects (Yin, 1969). The FIE has been suggested to result from a substantial difficulty to process configural or holistic information from inverted faces (Maurer et al., 2002; Rossion, 2008). Given that other-race faces are processed less holistically, one might assume that the FIE should be smaller for these faces, a finding which has indeed been observed repeatedly (e.g., Rhodes et al., 1989; Hancock and Rhodes, 2008). Moreover, it is known that face inversion affects the N170, which is increased and delayed for inverted relative to upright faces (e.g., Eimer, 2000; Rossion et al., 2000; Itier and Taylor, 2002). Accordingly, one might expect a larger N170 FIE for own- relative to other-race faces. However, results on this issue are also mixed, with some studies showing larger N170 inversion effects for own-race faces (Vizioli et al., 2010a; Caharel et al., 2011; Montalan et al., 2013), whereas others do not (Wiese et al., 2009; Chen et al., 2013). As can be seen in Table 1, the number of relevant studies is relatively small to date, but an enhanced N170 FIE for own-race faces has been shown repeatedly in categorization tasks, while the situation with more superficial tasks is less clear.

Finally, a number of ERP studies tested effects of face ethnicity on the amplitude of the occipito-temporal P2, a positive-going component subsequent to N170, which has been suggested to reflect the processing of second-order configurations (Mercure et al., 2008), i.e., the metric distances between facial features, or the typicality of a face relative to a prototype (Schulz et al., 2012). Previous studies have reported substantially larger P2 amplitudes at both left- and right-hemispheric electrode sites for own- relative to other-race faces in participants without particular expertise for other-race faces (Stahl et al., 2008; Lucas et al., 2011), whereas participants with substantial contact to people from the other ethnic background showed only small ethnicity effects in the P2 (Stahl et al., 2008; Wiese et al., 2013). Importantly for the present purpose, the P2 effect has also been found to be modulated by task demands (Stahl et al., 2010), as it was substantially smaller and restricted to right-hemispheric electrodes when participants were asked to rate own- and other-race faces for attractiveness as compared to categorizing them according to ethnicity. However, it is unclear whether the higher amount of facial detail necessary for the successful completion of the attractiveness task or the reduced salience of ethnicity information in this condition led to the reduced P2 effect.

The present study aimed at testing the following predictions. (i) If the presence vs. absence of the N170 ethnicity effect for upright faces depended on task demands, this should be detectable using a within-subjects manipulation, which excludes the possibility of confounds by uncontrolled group variables. N170 amplitude should be similar for own- and other-race faces in a more superficial task, in which detailed facial information is not task-relevant (orientation task). By contrast, N170 amplitudes should be larger for other-race faces when category information is task-relevant (ethnicity task). Only few studies on categorization tasks are available, and the present study aimed at adding further evidence to this least often tested task category^3^. (ii) A larger N170 FIE for own-race faces has been observed in tasks which either emphasized the processing of detailed facial information (Caharel et al., 2011; Montalan et al., 2013) or not (Vizioli et al., 2010a). Moreover, two further studies with superficial tasks did not find a larger FIE for own-race faces (Wiese et al., 2009; Chen et al., 2013). I thus expected to find an increased N170 FIE for own-race faces in the ethnicity task, whereas it was less clear whether a corresponding effect would emerge in the more superficial orientation task or not. (iii) If the previously observed reduction of the P2 ethnicity effect in an attractiveness judgment relative to an ethnicity categorization task was related to the larger amount of detailed face processing required for the attractiveness decision, a more superficial orientation task should result in a similar (or even larger) P2 effect as a categorization task. If, however, salience of ethnicity information contributes to the P2 effect, it should be larger in the categorization relative to the orientation task. Finally, to test whether any potential task effects were selective for faces, I added non-facial control stimuli to the experiment (i.e., Asian and European houses).

## Methods

### Participants

Twenty right-handed Caucasian students from the University of Jena (13 female, mean age = 22.0 years ± 2.1 *SD*) contributed data. All participants reported normal or corrected-to-normal vision and no history of neurological or psychiatric disorders. Participants received course credits or a monetary reward of 5€/h for partaking. All participants gave written informed consent and the study was approved by the ethics committee of the Faculty of Social and Behavioral Sciences at Jena University.

### Stimuli

Color images depicting 50 Asian and 50 Caucasian full-frontal faces with neutral or moderately happy expressions (50% female respectively), as well as 50 Asian and 50 European houses were taken from various internet resources. The house stimuli consisted of traditional buildings only to ensure easy differentiation of their cultural origin. Faces and houses were cut out and pasted in front of a uniform black background, such that no clothing or background information was visible. All stimuli were cropped to a frame of 300 × 380 pixels, resulting in a visual angle of 6.7° × 8.5° at a viewing distance of 90 cm. Stimuli were matched for luminance and contrast using Adobe Photoshop. Inverted versions of all stimuli were created by picture-plane rotations of the images by 180°.

Five participants (all female, mean age = 22.2 years ± 2.8 *SD*), who did not take part in the main experiment, rated all face stimuli in upright orientation for emotional expressions on a 7-point scale (ranging from 1 = very angry to 7 = very happy; 4 = neutral). An item analysis showed that both Caucasian and Asian faces were rated as showing neutral expressions (Caucasian faces: *M* = 3.93 ± 0.71 *SD*; Asian faces: *M* = 4.29 ± 0.60 *SD*). At the same time, Asian faces were rated more happy than Caucasian faces [*t*_(98)_ = 2.74, *p* = 0.007, Cohen's *d* = 0.55].

### Procedure

Participants were seated in a dimly lit, electrically shielded and noise-attenuated cabin (400-A-CT-Special, Industrial Acoustics, Niederkrüchten, Germany) with their heads in a chin rest. The experiment consisted of two practice blocks (one for each of the two tasks) using additional stimuli and eight experimental blocks. Each trial started with the presentation of a fixation cross that randomly varied in duration between 1000 and 1500 ms, followed by the presentation of a face or house stimulus for 1000 ms. In different experimental blocks, participants were asked to indicate the orientation (upright, inverted) or the ethnic/cultural background of each stimulus (Asian or European). Participants were asked to respond via key presses using their left and right index fingers as quickly as possible without compromising accuracy. The assignment of keys to response categories was counterbalanced across participants.

The experimental design varied the factors stimulus type (face vs. house), ethnicity (Asian vs. European), orientation (upright vs. inverted), and task (orientation task, ethnicity task) within-subjects. The task changed after each block, and task order was balanced across participants (ABABABAB vs. BABABABA). Each block contained 100 trials with either 12 or 13 trials per condition, which were presented randomly intermixed. Each individual image was presented twice in the course of the experiment, once in the ethnicity and once in the orientation task.

After the main experiment, all participants completed a questionnaire (see Wiese, 2012) asking them to indicate the amount of contact they have with Asian and European people (in h/week), the number of contact persons (per week) from both ethnic groups, and the intensity of contact (0 = no contact, 1 = very superficial to 4 = very intense) with Asian and European people in daily-life situations (such as job/university, meeting friends/spare time activities, family, domestic circumstances). Total scores were calculated for each participant by summing up (h/week, number of persons/week) or averaging (contact quality) self-report measures from the different situations separately for Asian and European contacts.

### EEG recording and analysis

EEG was recorded using a 64-channel BioSemi Active II system (BioSemi, Amsterdam, Netherlands). Active sintered Ag/AgCl-electrodes were mounted in an elastic cap, and EEG was recorded continuously with a 512 Hz sampling rate from DC to 155 Hz. Note that BioSemi systems work with a “zero-ref” setup with ground and reference electrodes replaced by a so-called CMS/DRL circuit (cf. to http://www.biosemi.com/faq/cms&drl.htm for further information).

Blink artifacts were corrected using the algorithm implemented in BESA 5.3 (MEGIS Software GmbH, Graefelfing, Germany). EEG was segmented relative to stimulus onset from −200 to 1000 ms, with a 200 ms baseline. Trials contaminated by non-ocular artifacts and saccades were rejected using the BESA 5.3 tool, with an amplitude threshold of 100 μV and a gradient criterion of 75 μV. Remaining trials were re-calculated to average reference, averaged according to experimental condition and digitally low-pass filtered at 40 Hz (12 db/oct, zero phase shift).

Latency of early ERP components (P1, N170) was analyzed at the electrodes of their respective maximum and the respective contralateral homologue (O1/O2 for P1; P9/P10 for N170). P1 peak amplitude was measured at electrodes O1 and O2 in a time window from 90 to 140 ms, N170 peak amplitude was measured at P7/P8, PO7/PO8, P9/P10, and PO9/PO10 in a time window from 140 to 210 ms. For P2, mean amplitudes were analyzed at electrodes P7/P8, PO7/PO8, P9/P10, and PO9/PO10 from 210 to 300 ms. Separate repeated-measures analyses of variance (ANOVAs) were calculated for each component. When appropriate, degrees of freedom were corrected according to the Huynh-Feldt procedure.

## Results

### Contact questionnaire

Participants reported substantially more contact to European relative to Asian people [contact time: *M*_European_ = 52.4 h/week ± 27.1 *SD*, *M*_Asian_ = 3.0 h/week ± 7.5 *SD*, *t*_(19)_ = 7.76, *p* < 0.001, Cohen's *d* = 2.638; number of contact persons: *M*_European_ = 31.5 ± 22.9 *SD*, *M*_Asian_ = 1.0 ± 1.6 *SD*, *t*_(19)_ = 6.20, *p* < 0.001, Cohen's *d* = 1.879]. Moreover, participants indicated more intense contact to European relative to Asian people [*M*_European_ = 2.6 ± 1.3 *SD*, *M*_Asian_ = 0.7 ± 1.3 *SD*, *t*_(19)_ = 4.42, *p* < 0.001, Cohen's *d* = 1.385].

### Performance

**Figure 1:**
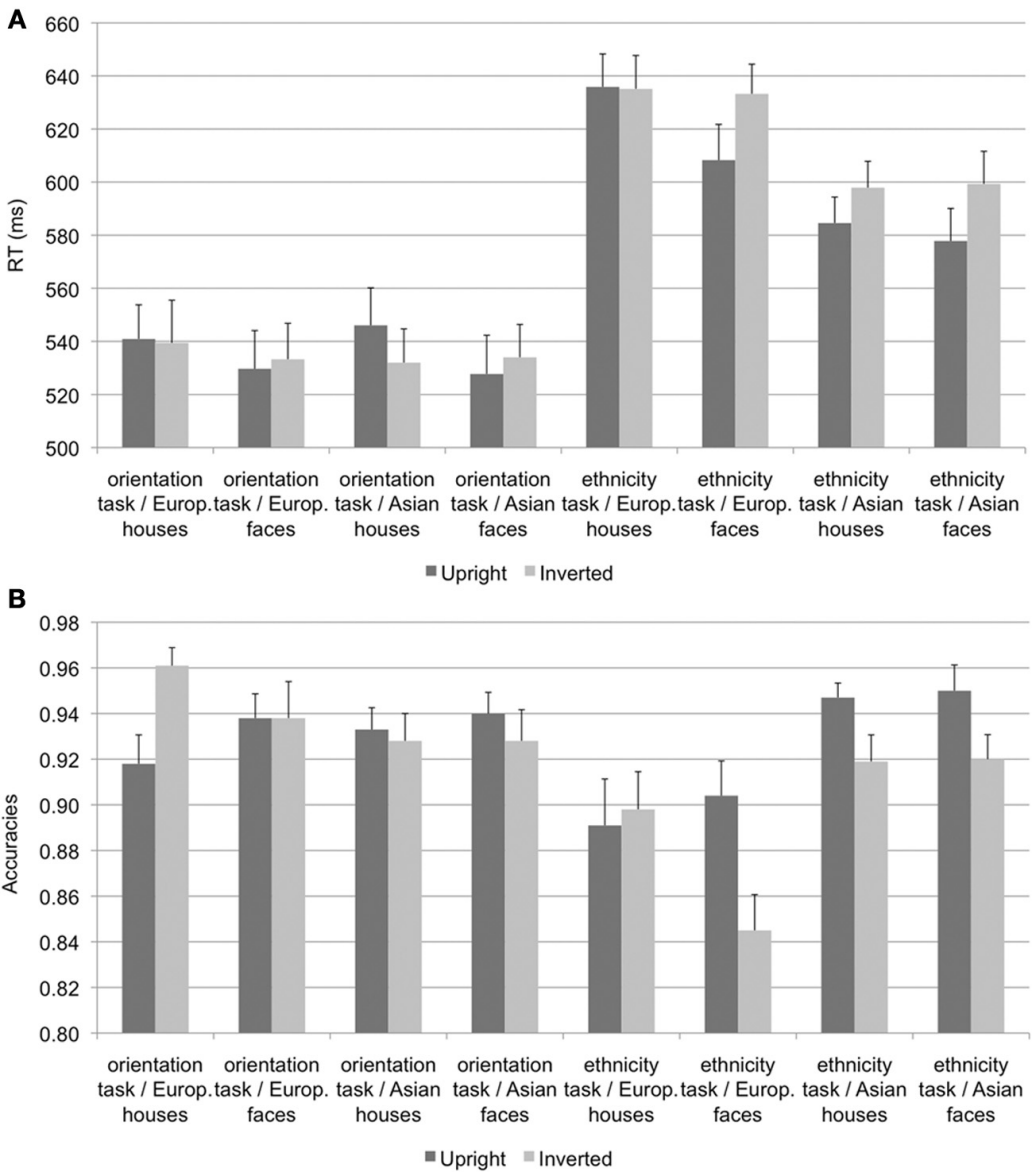
**Mean reaction times **(A)** and accuracies **(B)**, error bars denote standard errors of the mean**.

A repeated-measures ANOVA on mean reaction times for correct responses (see Figure 1A) with the within-subject factors task (orientation vs. ethnicity task), stimulus type (face vs. house), ethnicity (Asian vs. European), and orientation (upright vs. inverted) revealed significant main effects of task [*F*_(1, 19)_ = 114.98, *p* < 0.001, η^2^_*p*_ = 0.858], stimulus type [*F*_(1, 19)_ = 15.10, *p* = 0.001, η^2^_*p*_ = 0.443], ethnicity [*F*_(1, 19)_ = 28.73, *p* < 0.001, η^2^_*p*_ = 0.602], and orientation [*F*_(1, 19)_ = 5.15, *p* = 0.035, η^2^_*p*_ = 0.213]. These main effects were qualified by significant interactions of task × ethnicity [*F*_(1, 19)_ = 44.58, *p* < 0.001, η^2^_*p*_ = 0.701], reflecting faster responses for Asian stimuli in the ethnicity but not in the orientation task, task × orientation [*F*_(1, 19)_ = 5.58, *p* = 0.029, η^2^_*p*_ = 0.227], with faster responses for upright stimuli in the ethnicity but not in the orientation task, and stimulus type × orientation [*F*_(1, 19)_ = 16.57, *p* = 0.001, η^2^_*p*_ = 0.466], with faster responses to upright relative to inverted faces but not houses.

A corresponding analysis on accuracies (see Figure 1B) revealed significant main effects of task [*F*_(1, 19)_ = 11.53, *p* = 0.003, η^2^_*p*_ = 0.378], ethnicity [*F*_(1, 19)_ = 11.36, *p* = 0.003, η^2^_*p*_ = 0.374], and orientation [*F*_(1, 19)_ = 5.94, *p* = 0.025, η^2^_*p*_ = 0.238], as well as significant two-way interactions of task × ethnicity [*F*_(1, 19)_ = 15.10, *p* = 0.001, η^2^_*p*_ = 0.443], with more accurate responses for Asian than European stimuli in the ethnicity but not in the orientation task, and task × orientation [*F*_(1, 19)_ = 13.84, *p* = 0.001, η^2^_*p*_ = 0.422], with more accurate responses for upright relative to inverted stimuli in the ethnicity but not in the orientation task. Finally, an interaction of stimulus type × orientation [*F*_(1, 19)_ = 15.42, *p* = 0.001, η^2^_*p*_ = 0.448] was further qualified by a three-way interaction of ethnicity × stimulus type × orientation [*F*_(1, 19)_ = 10.30, *p* = 0.005, η^2^_*p*_ = 0.352]. Follow-up analyses revealed significant inversion effects, with more accurate responses for upright than inverted stimuli for European faces [*F*_(1, 19)_ = 6.79, *p* = 0.017, η^2^_*p*_ = 0.263], Asian faces [*F*_(1, 19)_ = 6.99, *p* = 0.016, η^2^_*p*_ = 0.269], and Asian houses [*F*_(1, 19)_ = 5.26, *p* = 0.033, η^2^_*p*_ = 0.217], but more accurate responses for inverted relative to upright European houses [*F*_(1, 19)_ = 6.14, *p* = 0.023, η^2^_*p*_ = 0.244].

### Event-related potentials

**Figure 2:**
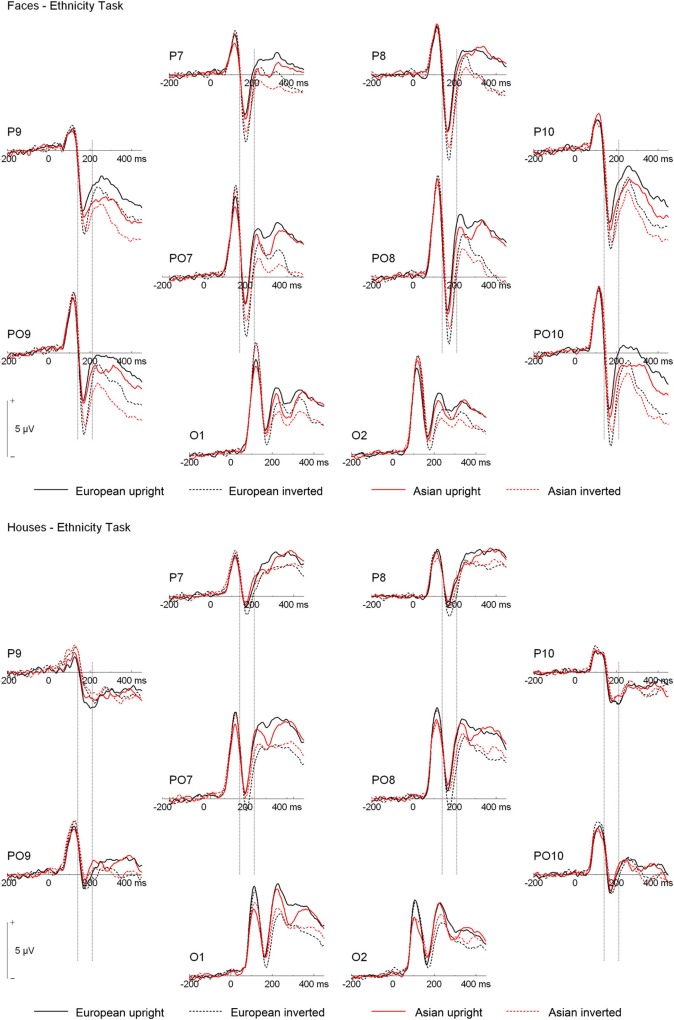
**Grand mean ERPs from the ethnicity task. Vertical lines indicate the N170 time window**.

**Figure 3:**
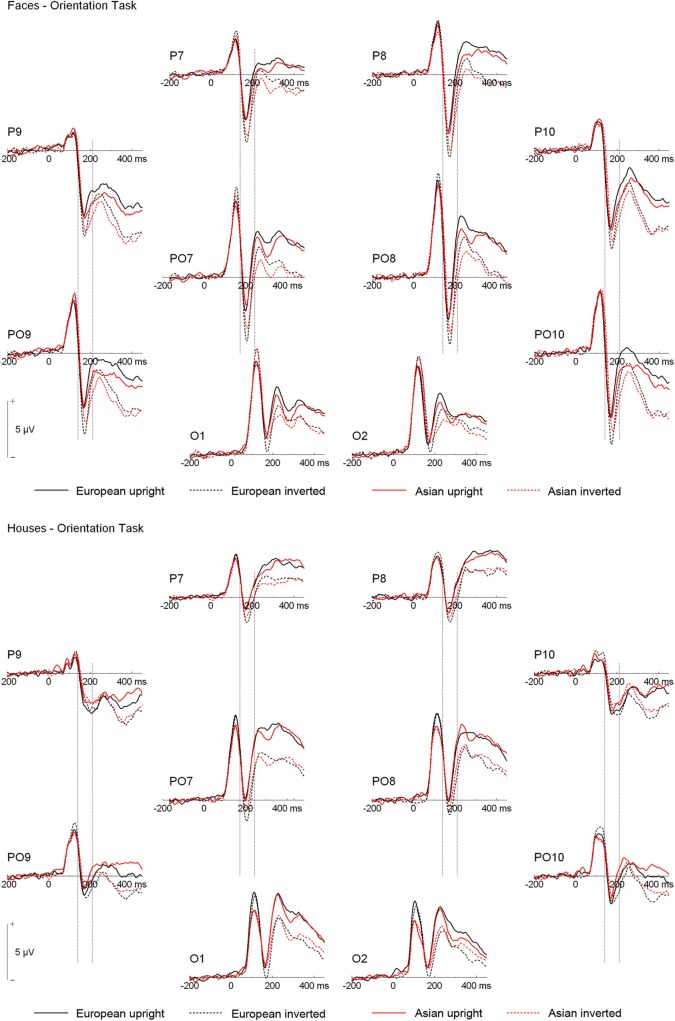
**Grand mean ERPs from the orientation task. Vertical lines indicate the N170 time window**.

ERPs are depicted in Figures 2 and 3. The number of trials per condition in an individual participant included in the statistical analyses ranged from 27 to 50 (*M* = 44 ± 5 *SD*). In the following paragraphs, main effects of hemisphere or site, as well interactions containing only those factors are not reported. In addition, main effects and interactions qualified by higher-order interactions are not described in the text. A complete list of all significant effects and all statistical indices for the omnibus tests can be found in Table 2.

**Table 2:** **Significant effects of omnibus ANOVAs on ERP data**.

	***F***	***df***	***p***	**η^2^_*p*_**
**P1 PEAK AMPLITUDE**				
Hemisphere	7.96	1, 19	0.011	0.295
Ethnicity	25.69	1, 19	<0.001	0.575
Stimulus type	30.59	1, 19	<0.001	0.617
Orientation	15.70	1, 19	<0.001	0.452
Ethnicity × stimulus type	19.28	1, 19	<0.001	0.504
Hemisphere × ethnicity × stimulus type	4.79	1, 19	0.041	0.201
Stimulus type × orientation	23.96	1, 19	<0.001	0.558
**P1 PEAK LATENCY**				
Ethnicity	4.94	1, 19	0.039	0.206
Stimulus type	21.63	1, 19	<0.001	0.532
Orientation	12.96	1, 19	0.002	0.406
**N170 PEAK AMPLITUDE**				
Site	25.15	3, 57	<0.001	0.570
Task	9.30	1, 19	0.007	0.329
Ethnicity	8.05	1, 19	0.011	0.297
Stimulus type	122.56	1, 19	<0.001	0.866
Orientation	33.04	1, 19	<0.001	0.635
Site × task	4.11	3, 37	0.010	0.178
Task × stimulus type	10.01	1, 19	0.005	0.345
Site × ethnicity × stimulus type	3.13	3, 57	0.033	0.141
Site × orientation	13.42	3, 57	<0.001	0.414
Ethnicity × orientation	11.84	1, 19	0.003	0.384
Site × ethnicity × orientation	5.96	3, 57	0.001	0.239
Stimulus type × orientation	11.15	1, 19	0.003	0.370
Site × stimulus type × orientation	9.01	3, 57	<0.001	0.322
Site × ethnicity × stimulus type × orientation	3.12	3, 57	0.033	0.141
**N170 PEAK LATENCY**				
Hemisphere	5.19	1, 19	0.034	0.215
Stimulus type	41.20	1, 19	<0.001	0.684
Orientation	17.67	1, 19	<0.001	0.482
Ethnicity × stimulus type	18.04	1, 19	<0.001	0.487
**P2 MEAN AMPLITUDE**				
Site	100.36	3, 57	<0.001	0.841
Ethnicity	29.09	1, 19	<0.001	0.605
Stimulus type	43.68	1, 19	<0.001	0.697
Orientation	54.28	1, 19	<0.001	0.741
Task × ethnicity	4.76	1, 19	0.042	0.200
Site × task × ethnicity	5.82	3, 57	0.002	0.235
Task × stimulus type	5.82	1, 19	0.026	0.234
Ethnicity × stimulus type	60.86	1, 19	<0.001	0.762
Hemisphere × task × ethnicity × stimulus type	4.93	1, 19	0.039	0.206
Stimulus type × orientation	5.64	1, 19	0.028	0.229
Task × stimulus type × orientation	5.35	1, 19	0.032	0.220

P1. A repeated-measures ANOVA on P1 peak amplitude with the factors hemisphere (left vs. right), task, stimulus type, ethnicity and orientation revealed a significant interaction of stimulus type × orientation, reflecting similar amplitudes for inverted relative to upright houses, but larger amplitudes for inverted relative to upright faces. Additionally, an interaction of hemisphere × ethnicity × stimulus type reflected larger amplitudes for European relative to Asian houses but not faces, an effect which was slightly larger at electrode O2.

Analysis of P1 latencies at O1 and O2 revealed significant main effects of stimulus type, reflecting earlier P1 peaks for houses than faces, ethnicity, with slightly earlier peaks for European stimuli, and orientation, with slightly earlier peaks for upright than inverted stimuli.

**Figure 4:**
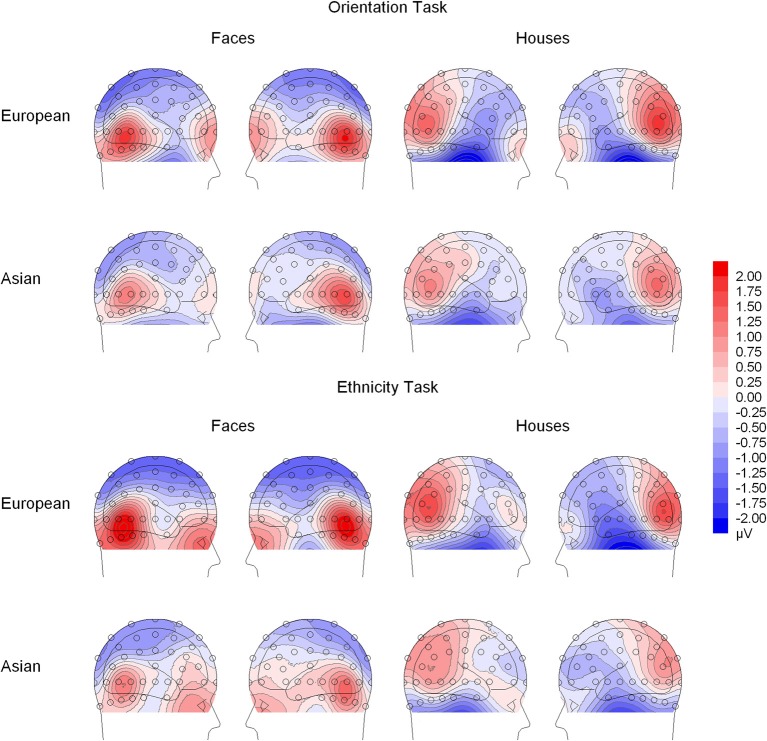
**Scalp-topographical voltage maps (spherical spline interpolation, 90° equidistant projection) depicting the inversion effect (upright—inverted conditions) in the N170 time window**.

N170. Analysis of N170 peak amplitude yielded a significant interaction of task × stimulus type, reflecting larger amplitudes for houses in the orientation than ethnicity task. Moreover, an interaction of site × ethnicity × stimulus type × orientation was observed. Follow-up tests for face stimuli revealed significant interactions of ethnicity × orientation, reflecting larger inversion effects for European relative to Asian faces, at electrodes PO9/PO10 [*F*_(1, 19)_ = 18.87, *p* = 0.003, η^2^_*p*_ = 0.385], P9/P10 [*F*_(1, 19)_ = 10.50, *p* = 0.004, η^2^_*p*_ = 0.356], P7/P8 [*F*_(1, 19)_ = 5.89, *p* = 0.025, η^2^_*p*_ = 0.237], and PO7/PO8 [*F*_(1, 19)_ = 6.41, *p* = 0.020, η^2^_*p*_ = 0.252; see Figure 4]. For houses a significant interaction of ethnicity × orientation was detected at PO7/PO8 [*F*_(1, 19)_ = 10.76, *p* = 0.004, η^2^_*p*_ = 0.362], with larger inversion effects for European relative to Asian houses, but not at any of the other electrode sites (0.16 < *F_s_* < 4.06, 0.058 < *p_s_* < 0.898). The interactions of task × stimulus type × ethnicity [*F*_(1, 19)_ = 0.56, *p* = 0.464, η^2^_*p*_ = 0.029] and task × stimulus type × ethnicity × orientation [*F*_(1, 19)_ = 0.34, *p* = 0.569, η^2^_*p*_ = 0.017] were not significant in the omnibus ANOVA.

As the substantial between-category effects in N170 (i.e., houses vs. faces) may have obscured more subtle within-category effects (i.e., Asian faces vs. Caucasian faces) in the analyses described above, an additional ANOVA was carried out, in which only faces were used (see Wiese et al., 2009 for a similar approach). This analysis revealed significant main effects of site [*F*_(3, 57)_ = 15.89, *p* < 0.001, η^2^_*p*_ = 0.455] and orientation [*F*_(1, 19)_ = 30.19, *p* < 0.001, η^2^_*p*_ = 0.614], as well as significant two-way interactions of site × orientation [*F*_(3, 57)_ = 2.60, *p* = 0.043, η^2^_*p*_ = 0.151], and ethnicity × orientation [*F*_(1, 19)_ = 10.35, *p* = 0.005, η^2^_*p*_ = 0.353], reflecting larger inversion effects for Caucasian relative to Asian faces. Importantly, the five-way interaction of hemisphere × site × task × race × orientation was significant [*F*_(3, 57)_ = 2.91, *p* = 0.042, η^2^_*p*_ = 0.133]. *Post-hoc t-tests* were calculated to see whether N170 amplitude differed between Asian and Caucasian faces, but I restricted these analyses to those electrode sites that had shown ethnicity effects in previous studies (P9/P10, PO9/PO10; see e.g., Wiese et al., 2013). For upright faces, N170 in the ethnicity task was larger for Asian relative to Caucasian faces at both P9 [*t*_(19)_ = 2.27, *p* = 0.035, Cohen's *d* = 0.274]^4^ and PO10 [*t*_(19)_ = 2.28, *p* = 0.034, Cohen's *d* = 0.180], but neither at PO9 [*t*_(19)_ = 0.98, *p* = 0.339, Cohen's *d* = 0.108] or P10 [*t*_(19)_ = 1.34, *p* = 0.197, Cohen's *d* = 0.119]. In the orientation task, no significant differences between upright Asian and Caucasian faces were detected [0.69 < *t_s_* < 1.94, 0.067 < *p_s_* < 0.499]. For inverted faces, no significant differences were observed in the ethnicity categorization task (0.17 < *t_s_* < 1.32, 0.203 < *p_s_* < 0.864), whereas in the orientation task N170 was larger for Caucasian faces at PO10 [*t*_(19)_ = 3.07, *p* = 0.006, Cohen's *d* = 0.226], but not at P9 [*t*_(19)_ = 1.35, *p* = 0.193, Cohen's *d* = 0.134], PO9 [*t*_(19)_ = 1.99, *p* = 0.061, Cohen's *d* = 0.208], or P10 [*t*_(19)_ = 1.39, *p* = 0.179, Cohen's *d* = 0.155]. Thus, whereas ethnicity effects in N170 for upright faces were only evident in the ethnicity task (with larger amplitudes for other-race faces), ethnicity effects for inverted faces were only observed in the orientation task (with larger amplitudes for own-race faces). Finally, two ANOVAs were conducted to confirm that larger inversion effects for own-race relative to other-race faces occurred in both tasks. The critical interaction of ethnicity × orientation was significant both in the orientation [*F*_(1, 19)_ = 7.73, *p* = 0.012, η^2^_*p*_ = 0.289] and in the ethnicity categorization task [*F*_(1, 19)_ = 5.30, *p* = 0.033, η^2^_*p*_ = 0.218].

Analysis of N170 latency at P9/P10 revealed significant main effects of stimulus type, with delayed peaks for houses relative to faces, and orientation, reflecting delayed peaks for inverted relative to upright stimuli (see Table 2). Moreover, an interaction of ethnicity × stimulus type was observed, with delayed N170 responses for Asian relative to European faces [*F*_(1, 19)_ = 34.28, *p* < 0.001, η^2^_*p*_ = 0.643], but not houses [*F*_(1, 19)_ = 2.60, *p* = 0.123, η^2^_*p*_ = 0.120].

P2. A repeated-measures ANOVA on P2 amplitude yielded significant interactions of ethnicity × stimulus type, with larger amplitudes for European relative to Asian faces but not houses, and site × task × ethnicity, reflecting particularly pronounced ethnicity effects in the categorization task at P9/P10. A further interaction of task × stimulus type × orientation was indicative of a larger inversion effect for houses in the orientation compared to the ethnicity task, whereas faces elicited similar inversion effects in the two tasks. Finally, a significant interaction of hemisphere × task × ethnicity × stimulus type was detected. *Post-hoc* analyses for face stimuli revealed significantly larger ethnicity effects over the left hemisphere in the ethnicity relative to the orientation task [interaction of ethnicity × task: *F*_(1, 19)_ = 5.59, *p* = 0.029, η^2^_*p*_ = 0.227], whereas ethnicity effects over the right hemisphere did not interact with task (*F* < 1). At the same time, European faces elicited significantly more positive amplitudes than Asian faces in both tasks and over both the left and right hemisphere, which was reflected in significant main effects of ethnicity in all possible combinations of these factors [all *F*_(1, 19_) > 27, all *p* < 0.001, all η^2^_*p*_ > 0.592]. *Post-hoc* tests for house stimuli revealed no significant ethnicity effects, neither in the ethnicity nor in the orientation task (all *p* > 0.1).

## Discussion

The present study tested the effect of task demands on the neural processing of own- (i.e., European) and other-race (i.e., Asian) faces and non-facial control stimuli (i.e., houses). Concerning the predictions outlined in the introduction, the following main results can be summarized: First, task demands affected the N170 ethnicity effect for upright faces. More specifically, the ERP analysis revealed a larger N170 for upright other- relative to own-race faces in the categorization task, but no such effect in the orientation task. Second, a larger N170 FIE was observed for own- relative to other-race faces. This interaction of face ethnicity and orientation generalized across tasks. Finally, the P2 ethnicity effect observed over the left hemisphere was reduced in the orientation relative to the ethnicity task, suggesting that a high saliency of ethnicity information amplifies this effect, but is not an essential prerequisite for its emergence. These ERP results and additional behavioral findings are discussed in the following paragraphs.

Most importantly, the analysis of ERP data revealed larger N170 amplitudes for upright other- relative to own-race faces in the ethnicity categorization task but not in the orientation task. This finding is in line with the literature reviewed in the introduction (see Table 1), in which the majority of studies using superficial tasks found no N170 ethnicity effect, whereas the majority of studies using categorization or identity tasks found larger N170 amplitudes for other-race faces. Similarly, a recent study by Senholzi and Ito (2013) observed no N170 ethnicity effect in a superficial task and larger amplitudes for other-race faces in an identity task. The present experiment adds to this previous finding by showing that even tasks that do not explicitly require the processing of identity information result in N170 ethnicity effects. It may therefore be seen as a stronger test to the idea that the N170 ethnicity effect is not elicited by particularly superficial tasks. At variance with the present findings, however, Senholzi and Ito (2013) reported larger amplitudes for *own*-race than other-race faces in a categorization task. This pattern has not been reported previously, with the exception of one study (Ito and Urland, 2005), which used an average mastoid reference potentially obscuring any experimental effects at the nearby T5/T6 electrodes where N170 was measured. While the exact reason for this discrepancy remains unclear, the present results are in line with the majority of studies using categorization tasks.

It should be noted that the larger N170 for upright other- relative to own-group faces seems quite specific to face ethnicity, and does not reflect a more general mechanism differentiating between any social in-group vs. out-group faces. For instance, while the N170 ethnicity effect occurs in both Asian and European participants (Wiese et al., 2013), a similar interaction of stimulus category by participant group with larger amplitudes for other-group faces is not observed for own- vs. other-age or own- vs. other-gender faces (see e.g., Wiese et al., 2008, 2012b; Melinder et al., 2010; Wolff et al., 2013). In sum, the present findings support the idea that ethnicity effects in N170 amplitude critically depend on task demands.

Interestingly, although N170 was larger for inverted own-race faces in the orientation task only, the N170 FIE was larger for own-race faces in both tasks. The N170 inversion effect has been suggested to reflect perceptual expertise for a given class of stimuli (Rossion et al., 2002), and larger effects for own-race faces are therefore well in line with expertise accounts of the own-race bias (e.g., Tanaka et al., 2004; Rossion and Michel, 2011). The present finding is also in line with those previous studies that observed a larger N170 FIE for own-race faces (Vizioli et al., 2010a; Caharel et al., 2011), but not with others that did not (Wiese et al., 2009; Chen et al., 2013). While the reason for this discrepancy remains somewhat unclear, it may be related to the fact that Chen et al. (2013) tested exclusively Chinese participants. Recent evidence suggests that Asian participants show a similar degree of holistic processing for own- and other-race faces (Crookes et al., 2013), possibly reflecting a larger degree of variability tolerated by the face processing system of this participant group. Moreover, it has been suggested that the presence of non-face stimuli in our previous study (Wiese et al., 2009) may have affected the results, as those previous studies showing a larger own-race FIE did not use additional non-face stimuli (Caharel et al., 2011). This suggestion is not supported by the present experiment, which demonstrated a corresponding result even though house stimuli were randomly intermixed in all conditions.

The typical finding that N170 amplitudes are larger for inverted relative to upright faces has been explained by Itier et al. (2007) and Itier and Batty (2009) by suggesting that N170 to upright faces reflected the activation of face-sensitive neurons, whereas N170 to inverted faces was elicited by the combined activity of both face- and eye-sensitive neurons (see also Kloth et al., 2013). When upright faces are presented, activity of eye cells is inhibited. This framework offers a possible explanation of the present results (see Table 3), as the larger N170 for upright other-race relative to upright own-race faces may reflect less efficient inhibition of eye cells for other-race faces. At the same time, larger N170 amplitudes for inverted own-race faces may result from the fact that the eye region for faces from different ethnic groups substantially differs, and that eyes of other-race faces may not be able to elicit eye cell activity to the same extent as own-race faces. Importantly, the efficiency of inhibition may to some degree depend on top-down modulations such as task demands. A task that requires more in-depth face processing (such as a categorization task) may lead to a sharpening of neural processing to face cells, and thus to strong inhibition of eye cells. This sharpening, however, may be possible only for own-race faces as these are more prototypical. In the orientation task, neural processing may not be tuned as specifically toward face cells, resulting in an incomplete (and similar) inhibition of eye cells for upright own- and other-race faces. Moreover, for inverted faces eye cell activity may reach the respective maximum possible level for both own- and other-race faces, resulting in larger N170 amplitudes for inverted own-race faces. Importantly, this interpretation suggests that eye rather than face cells are responsible for ethnicity effects in N170. Please note, however, that this interpretation of the present results is speculative and needs further testing in future studies.

**Table 3:** **A potential mechanism for N170 ethnicity effects**.

		**Ethnicity task**		**Orientation task**	
		**Face cells**	**Eye cells**	**Face cells**	**Eye cells**
Upright	Own-race	+++	+++	+++	+++
	Other-race	+++	++	+++	++
Inverted	Own-race	+++	+++	+++	+++
	Other-race	+++	++	+++	++

*Black crosses indicate actual activity, gray crosses indicate maximum possible activity*.

Of note, inversion effects in the N170 time range were also observed for house stimuli. As can be seen in Figure 4, these effects did not occur at sites where the N170 FIE was at maximum (P10/PO10) but had a clearly more dorsal scalp distribution, with largest effects occurring at PO7/PO8. This finding is in line with previous reports of inversion effects for houses at similar scalp positions (Eimer, 2000; Itier et al., 2006) and at lateral occipito-temporal intracranial electrodes (Rosburg et al., 2010). These findings may suggest that different neural populations elicit the house and face inversion effects. If so, these populations seem to respond differently under varying processing demands, as house inversion effects were larger in the orientation task, while FIEs were larger in the ethnicity task.

House inversion effects in the N170 time range were larger for European (“own-race”) relative to Asian (“other-race”) houses. Importantly, the larger FIE for own-race faces was detected at all tested electrode sites, whereas the enhanced effect for European houses was restricted to the more dorsal electrodes PO7/PO8. Generally in line with this finding, previous studies detected larger N170 amplitudes for objects of particular expertise relative to control stimuli at similar scalp sites (Tanaka and Curran, 2001). The present results suggest that larger inversion effects to own-culture stimuli are not completely face-selective. Instead, the more dorsal portion may reflect overall enhanced familiarity with own-culture stimuli, whereas the more ventral part may more selectively represent the fine-tuning of facial expertise. In sum, N170 inversion effects were observed to be larger for own- relative to other-race faces, which was independent of task and cannot be fully explained by generally enhanced familiarity with European stimuli. Instead, the larger N170 FIE for own-race faces appears to be at least partly related to the fine-tuning of processes selective for face stimuli.

Subsequent to N170, larger P2 amplitudes were observed for own- relative to other-race faces. This P2 ethnicity effect was modulated by task, with larger effects in the categorization relative to the orientation task, which is reminiscent of a previous study from our group (Stahl et al., 2010). In this previous experiment, we observed clearly bilateral P2 effects in an ethnicity categorization task, but only a small and right-lateralized effect in an individualization task, in which participants had to rate each of the faces for attractiveness. The finding in the present study, in which a reduced P2 effect in a superficial orientation task relative to a categorization task was observed, suggests that it is not the amount of facial detail necessary for a given task that affects the magnitude of the P2 ethnicity effect. Instead, it appears that the explicit processing of ethnicity information boosts the P2 effect over the left hemisphere. The right-hemispheric effect seems less affected by task demands, but is reduced by long-term expertise with other-race faces (Stahl et al., 2008). In contrast to N170, however, P2 effects were not correlated with the own-race bias in memory in a recent study (Wiese et al., 2013), and the role of P2 during the processing of own- and other-race faces therefore remains somewhat unclear.

In a recent study, Balas and Nelson (2010) presented own- and other-race faces with either consistent shape and pigmentation information (own-race shape + pigmentation, other-race shape + pigmentation) or inconsistent information (own-race shape/other-race pigmentation, other-race shape/own-race pigmentation). In a time window similar to the P2 in the present study (230–300 ms) the authors observed larger amplitudes for own- relative to other-race shape information. Interestingly, pigmentation information was observed to have the opposite effect, with more positive amplitudes for other-race pigmentation. These findings suggest that the P2 effects observed in the present and previous studies from our group (Stahl et al., 2008, 2010; Wiese et al., 2013) largely reflect differences in shape. It is noteworthy in this context that we used Asian and Caucasian own- and other-race faces, whereas Balas and Nelson (2010) used Caucasian and African-American faces. It is thus likely that pigmentation differences between own- and other-race faces were more pronounced in the latter study and relatively less perceptually salient in the present and our previous experiments.

It should be noted that each individual stimulus was presented twice in the present experiment, and it is thus possible that participants recognized some of the previously presented faces, even though the processing of individual identity was never task-relevant. One might suggest that the recognition of repeated faces may have been stronger in the categorization task, in which more detailed facial information was processed. Consequently, larger P2 ethnicity effects in the categorization relative to the orientation task may have not been related to ethnicity categorization *per se* but to more pronounced recognition of repeated faces. Although I cannot definitely exclude this possibility on the basis of the present data, it does not appear parsimonious when results of previous experiments are taken into account. If larger ethnicity effects in P2 were related to face recognition, this would suggest that in our previous study (Stahl et al., 2010) identity processing was stronger in an ethnicity categorization compared to an attractiveness rating task. This latter task, however, presumably required stronger processing of individual face information than the ethnicity task. In sum, while the suggestion that recognition of repeated faces enhanced the P2 effect in the present study appears less parsimonious than the alternative interpretation of stronger effects in case of ethnicity categorizations, further studies that avoid face repetition would be needed to definitely decide this question.

In addition to these ERP findings, two aspects of the behavioral results appear noteworthy. First, participants were faster to make ethnicity decisions for other-race than own-race stimuli. Similar findings have been reported by a number of previous studies, and have been interpreted to reflect the fast detection of an out-group defining feature in other-race faces (Levin, 1996, 2000). According to socio-cognitive theories of the own-race bias, this categorization advantage resulted in increased attention to general category compared to individuating information in other-race faces, which in turn led to less accurate memory (Hugenberg et al., 2010). The present results demonstrate that such a categorization advantage is not restricted to faces, but can also be observed for houses from a different culture. This general categorization advantage for “other-race” stimuli suggests that it is not face-selective, but may reflect an effect of overall familiarity extending to various stimulus classes.

Second, while no inversion effect was observed in the RT data of the orientation task, inversion slowed down participants' responses in the ethnicity task. Considering that the FIE is typically interpreted to reflect the disturbance of configural or holistic face processing (Rossion, 2008), this finding indicates that the categorization of facial ethnicity is not solely based on feature processing (for a recent demonstration of inversion effects in other categorization tasks, see Wiese et al., 2012a). This is at some variance with socio-cognitive accounts suggesting that the detection of race-specifying features drives ethnicity categorizations (Levin, 2000).

Finally, a potential limitation of the present study may be seen in the finding that Asian faces showed slightly but significantly happier expressions than Caucasian faces. Accordingly, this difference in expression may in principle have affected the present results, and may have led for instance to an increased N170 for happy rather than other-race faces. From my perspective, this assumption is not particularly likely for the following reasons: First, as noted above, a recent study from our group that used a similar face set found that effects of face ethnicity in the N170 and P2 interacted with participant ethnicity, and that both Caucasian and Asian participants demonstrated larger N170 amplitudes for the respective other-race category (Wiese et al., 2013). It is hard to see how this finding could be explained in terms of happier expressions in Asian faces. Second, a previous study did not detect differences in N170 amplitude for upright happy vs. neutral faces (Ashley et al., 2004). It should be further noted that this previous experiment used clearly emotional faces whereas in the present study, although a significant difference was detected, both Asian and Caucasian faces were rated as neutral on average.

In conclusion, the present results support the idea that differential processing of own- vs. other-race faces at early perceptual processing stages is modulated by task demands. More specifically, the necessity to process faces at a categorical or individual level appears to result in a larger N170 for own-race than other-race faces, while the processing of more superficial stimulus properties does not. At the same time, the N170 FIE is substantially larger for own-race than other-race faces in both tasks. This latter finding can only partly be explained by larger overall familiarity with more commonly seen stimuli and may thus reflect the fine-tuning of early perceptual processing stages to faces of maximum expertise.

### Conflict of interest statement

The author declares that the research was conducted in the absence of any commercial or financial relationships that could be construed as a potential conflict of interest.

## Footnotes

^1^ The term “race” is exclusively used to refer to visually distinct ethnic groups.

^2^Please note that I do not consider the experiments by Ito and Urland (2005) in this review, as N170 is unusually small in this study, presumably due to the untypical use of an average mastoid reference.

^3^I chose to not test all three tasks, as this would have made the experiment inappropriately long.

^4^Please note that the t tests in this section are not corrected for multiple comparisons, as they were calculated to clarify the significant interaction in the ANOVA.
